# Ethnic Prevalence of Angiotensin-Converting Enzyme Deletion (D) Polymorphism and COVID-19 Risk: Rationale for Use of Angiotensin-Converting Enzyme Inhibitors/Angiotensin Receptor Blockers

**DOI:** 10.1007/s40615-020-00853-0

**Published:** 2020-09-08

**Authors:** Rangaprasad Sarangarajan, Robert Winn, Michael A. Kiebish, Chas Bountra, Elder Granger, Niven R. Narain

**Affiliations:** 1 BERG LLC, 500 Old Connecticut Path, Bldg B, 3r Floor, Framingham, MA 01701 USA; 2 Massey Cancer Center, Virginia Commonwealth University, Richmond, VA 23298 USA; 3 Center for Medicines Discovery, Nuffield Department of Clinical Medicine, University of Oxford, Oxford, OX37DQ UK

**Keywords:** Angiotensin, ACE, Polymorphism, Ethnic, COVID-19

## Abstract

**Rationale:**

Hypertension, obesity and diabetes are major risk factors associated with morbidities underlying COVID-19 infections. Regression analysis correlated presence of ACE insertion/deletion (I/D) polymorphism to COVID-19 incidence and mortality. Furthermore, COVID-19 prevalence correlated to allele frequency of angiotensin-converting enzyme (ACE) deletion (D) polymorphism within the European population.

**Objective:**

Homozygous ACE deletion polymorphism is associated with increase in ACE and angiotensin II (Ang-II), sustained levels can result in inflammation, fibrosis and organ damage. The ACE DD polymorphism is also associated with hypertension, acute respiratory distress and diabetic nephropathy, all considered high risk for COVID-19 infection and outcomes. The study objective was to describe a biological framework associating ethnic prevalence of ACE deletion polymorphism to COVID-19 comorbidities providing rationale for therapeutic utility of ACE-I/ARBs to improve outcomes.

**Method and Results:**

The Allele Frequency Database (ALFRED) was queried for frequency of rs4646994 representing ACE I/D polymorphism. In a total of 349 worldwide population samples, frequency of ACE D allele was higher in European, Asian, and Africans cohorts. In the USA, the frequency of ACE D allele was higher in non-Hispanic Black compared with non-Hispanic White and Mexican Americans.

**Conclusion:**

COVID-19 binding mediated reduction/inactivation of ACE-II can increase ACE/Ang-II signalling pathway and related pathologies. The presence of ACE DD polymorphism with COVID-19 infection likely augments ACE/Ang-II activities, increasing severity of COVID-19 morbidities and impacts outcomes. Thus, ethnic prevalence of ACE DD polymorphism can explain in part the severity of COVID-19 morbidity providing rationale for the use of ACE-I/ARBs to improve outcomes.

## Introduction

The SARS-CoV-19 (COVID-19) infection has infected in excess of seventeen million individuals around the globe and is designated as a pandemic by the World Health Organization. The global efforts are focused on understanding the disease onset, progression and to identify causal linkage for differences in observed outcomes among the affected population and within specific demographics. Despite worldwide spread of the COVID-19 infections, European countries and the USA appear to have experienced higher incidence and mortality rates [1–3]. Hypertension, obesity, and diabetes were identified as the most common comorbidities associated with COVID-19 infection; higher severity of disease and mortality was generally reported in the elderly (> 50 years) population.

Angiotensin-converting enzyme 2 (ACE2) is the predominant receptor for SARS-CoV viral entry and infection, resulting in the reduction of expression of ACE2 [4, 5]. ACE2 is an enzyme component of the renin-angiotensin system (RAS), a complex integrated network of peptides-enzyme combination, generating catalytically active peptides with prominent influence on the vascular, renal, cardiac, and immune system [6]. In this report, we describe a framework of the pathophysiological consequence of COVID-19-induced reduction in ACE2, i.e., overactivation of the RAS pathway with the potential to have deleterious effect on organ functions including the lungs, kidneys, heart, and immune system. The deleterious activities of RAS within the COVID-19-infected cohorts can be further amplified by the presence of genetic polymorphism in the angiotensin-converting enzyme (ACE). Increased prevalence in frequency of the ACE polymorphism within ethnic groups, in part, is likely responsible for the observed severity of COVID-19 comorbidities and mortality in this population. This is substantiated by recent regression analysis linking presence of ACE-1 I/D (insertion/deletion) polymorphism with incidence and mortality with COVID-19 infection [7].

## Renin-Angiotensin System: ACE, Ang-II, and Inflammation

**Fig. 1:**
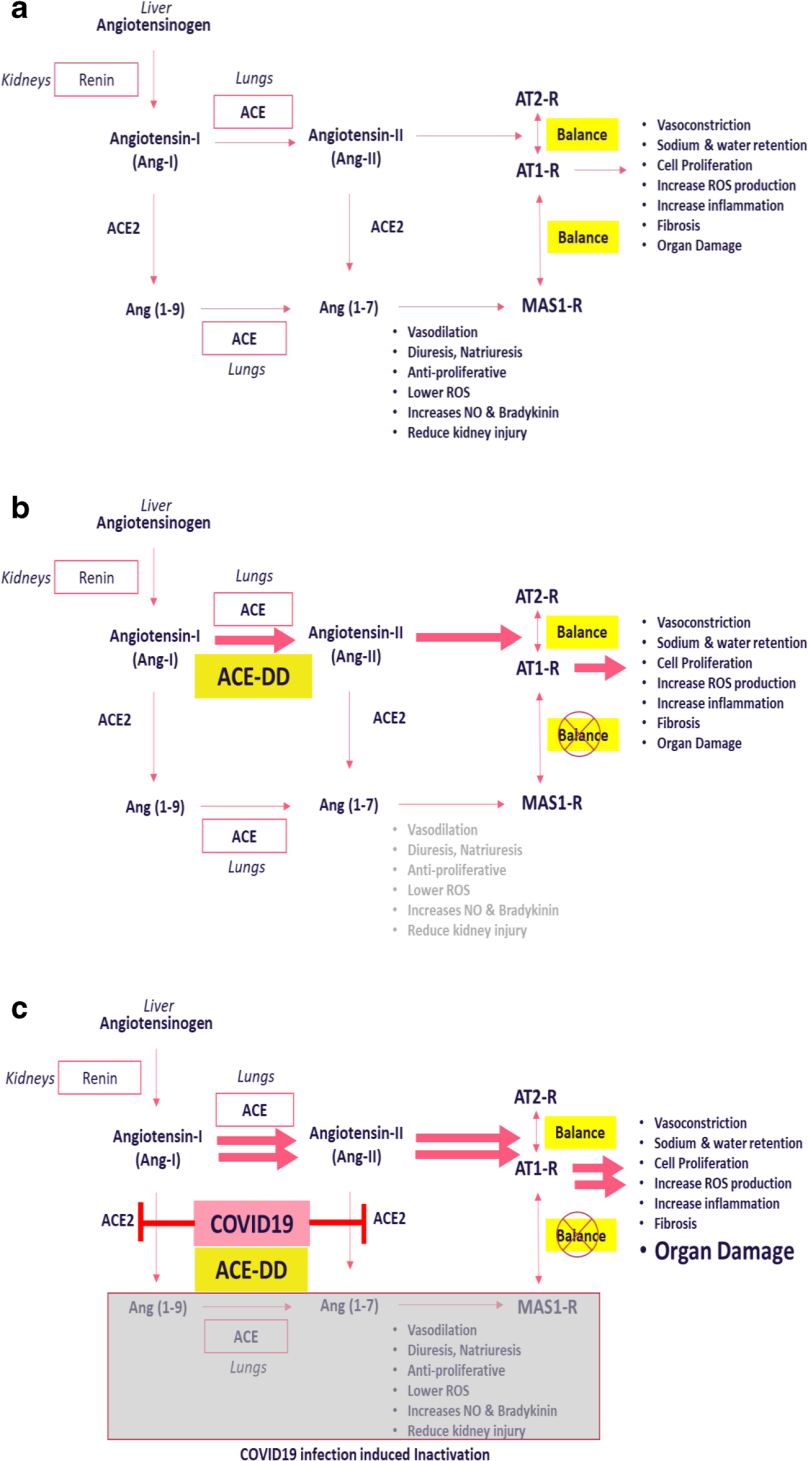
**a** Overview of the renin-angiotensin system. The figure describes the basic components of the renin angiotensin system with focus on the impact of ACE and ACE2 in the generation of angiotensin peptides, the respective cognate receptor(s) and corresponding physiological consequence of receptor activation. **b** Influence of ACE deletion (DD) polymorphism on renin-angiotensin system. The figure describes the consequence of the ACE deletion polymorphism, the increase in levels of ACE and angiotensin II resulting in activation of AT-1 receptor and downstream pathophysiological effects. **c** Consequence of COVID-19 infection and ACE Deletion (DD) polymorphism on renin-angiotensin system. The figure describes the increased activation of ACE and generation of Ang-II as a consequence of COVID-19-mediated reduction in ACE2 in the presence of ACE deletion polymorphism. The result is disruption of physiological balance of the ACE/ACE2 axis resulting in overactivation of AT1-R signalling and associated pathological consequence

The RAS system has a prominent role in the regulation of vascular dynamics; its components directly or indirectly influence functions of the lung, heart, kidney, brain and the immune system [6]. In addition to central RAS components, i.e., renin (kidney), ACE (lungs), and angiotensinogen (liver), tissue-specific localized systems including the kidney, heart, and lungs have been identified [6, 8]. Within RAS, the canonical angiotensin-converting enzyme (ACE) is responsible for conversion of angiotensin-1 (Ang-I) to angiotensin-2 (Ang-II) (Fig. 1a). Subsequently, Ang-II mediates its effects through activation of AT-1 and AT-2 receptors, resulting in distinct intracellular signalling pathways [9–11]. Activation of AT-1 receptors is associated with the well-characterized physiological actions of Ang-II in various organs including the lung, heart, kidney, and the vascular system [10].

In addition to its hemodynamic effect, Ang-II has significant pro-inflammatory effects, promoting generation of reactive oxygen species (ROS), cell proliferation, extracellular matrix remodelling, and regulation of gene expression via signalling pathways leading to tissue injury [8, 12]. Ang-II promotes expression of pro-inflammatory chemokines in the kidneys, heart, and vasculature to induce inflammation [13]. Several studies have characterized key inflammatory processes influenced by Ang-II on macrophages, dendritic cells, and mesangial cells resulting in mobilization and activation of cytokines, chemokines, and pro-inflammatory factors resulting in tissue damage and progressive organ failure [14]. Due to profound influence of Ang-II signalling pathways that are predominantly adverse when unmitigated, the potency of Ang-II is tightly regulated via proteolytic activities of enzymes to generate various angiotensin peptide fragments with physiological activities different from Ang-II [14] (Fig. 1a). ACE2 is an enzyme component of RAS, with proteolytic activities different from the canonical ACE. ACE2 is responsible for cleaving angiotensin I to Ang (1–9) and angiotensin-2 to Ang (1–7) peptides respectively (Fig. 1), of which the latter is a potent vasodilator [15, 16]. Several studies support a major role for Ang (1–7) in providing the counterbalance to the physiological effects of Ang-II [17–19]. Thus, the pro-inflammatory effects of ACE/Ang-II axis are balanced by activation of anti-inflammatory pathways by ACE2 and other systems.

## ACE Insertion/Deletion (ID) Polymorphisms: Prevalence

Two recent publications reported that ACE insertion/deletion polymorphism correlated to infectivity and mortality associated with COVID-19 infections [7, 20]. In humans, the gene encoding ACE is located on chromosome 17 and exhibits an insertion/deletion polymorphism that is characterized by an insertion (allele I) or deletion (allele D) of a 287 base pair marker in intron 16 that results in three different genotypes, i.e. DD or II homozygotes or ID heterozygotes. It is reported that the deletion (D) allele occurs in 55% of the population and associated with increased ACE activity, implicating the presence of D allele with disease pathologies associated with RAS activity [21].

The Allele Frequency Database (ALFRED; https://alfred.med.yale.edu/alfred/index.asp; RRID:SCR_001730) was queried for frequency of rs4646994 representing ACE I/D polymorphism, one of the best studies of all ACE polymorphisms. The allelic frequencies of the insertion (I, +) and deletion (D, −) genotypes within various geographic regions from 349 population samples were obtained from ALFRED and are summarized in Table 1. Inclusion of data from all European studies demonstrated almost equal distribution of the ACE (I) or ACE (D) allele, with Italians, Ashkenazi Jews and Canarians demonstrating slightly higher prevalence compared with the population averages. In contrast to Europe, among the African population, the frequency of D allele was almost twice compared with the I allele among 2126 population samples with highest levels observed in Pygmies, Ethiopian Jews, Moroccan, Nigerian and Tunisian populations. These are consistent with other studies reporting significant increase in the frequency of deletion polymorphism of ACE observed in individuals of African descent and associated with disease pathology [22]. Specifically, a prevalence of the D allele of 60% has been reported in individuals of African descent [22]. In the USA, the non-Hispanic Black population has higher frequency of the D allele (Table 2) compared with non-Hispanic White and Mexican American population [23]. The frequency of the D allele was increased compared with the I allele within the Middle Eastern population with higher values observed in both Arab and Saudi Arabia sample populations. In contrast to Africa and Middle East, increased frequency of the I allele was observed in sample populations from Asia (India, Pakistan Nepalese, Tajik regions and Sri Lanka), Oceania (New Zealand, Papua New Guinea and Micronesia), East Asia (China, Japan, Korea, Taiwan, Cambodia, Vietnam, Philippines and Malaysia) and South American countries.

**Table 1:** Prevalence of ACE insertion/deletion polymorphism: the Allele Frequency Database (ALFRED) was queried for identifying population frequency of the ACE insertion/deletion polymorphism among geographical locations. From a total of 349 population samples, the average frequencies of the insertion and deletion allele for ACE were calculated for the different geographical locations. The table provides the population sample size and frequency (italicized) and the breakdown of the frequency of the insertion and deletion allele within specific ethnic groups of interest within the population

		Sample size (*N*)	Insertion	Deletion
Europe		*16,220*	*0.412*	*0.588*
	Abazians	24	0.000	1.000
	Canarian	1358	0.375	0.625
	English	924	0.454	0.546
	French	2234	0.423	0.578
	Irish	226	0.429	0.571
	Italians	222	0.342	0.658
	Jews, Ashkenazi	154	0.340	0.660
Africa		*2126*	*0.340*	*0.660*
	Pygmies	68	0.221	0.779
	Jews, Ethiopian	64	0.203	0.797
	Moroccans	106	0.292	0.708
	Nigerians	22	0.273	0.727
	Tunisian	200	0.325	0.675
Middle East		*1714*	*0.360*	*0.640*
	Arabs	100	0.290	0.710
	Saudi	540	0.275	0.725
Asia		*7380*	*0.585*	*0.414*
Oceania		*1444*	*0.684*	*0.315*
East Asia		*3182*	*0.627*	*0.372*
South America		*2458*	*0.706*	*0.293*

**Table 2:** ACE polymorphism allele and genotype frequencies: the prevalence of 289-bp Alu insertion/deletion in intron 16 of ACE gene corresponding to rs4646994 within the non-Hispanic White and non-Hispanic Black population is described. (Information modified from source provided by Office of Science (OS), Office of Genomics and Precision Public Health, CDC 2009; complete data is available at https://www.cdc.gov/genomics/population/genvar/frequencies/ace.htm)

Gene variant	Race/ethnicity	Allele %		Allele % (95% CI)			Chi-square *p* Value	HW *p* Value
		D	I	DD	DI	II		
rs4646994	Non-Hispanic White	54.6	45.4	28.8 (25.9,31.8)	51.6 (47.8,55.3)	19.6 (17.7, 21.8)	< 0.001	0.11
	Non-Hispanic Black	58.7	41.3	33.8 (31.5,36.3)	49.8 (47.3,52.2)	16.4 (14.6,18.5)		0.1

## ACE Deletion (D) Polymorphism and Disease—Increased Susceptibility and Severity to Co-morbidities Associated with COVID-19

Although the ACE I/D polymorphism is located in a non-coding region, its presence is directly linked to regulation of renin-angiotensin system and associated pathological conditions. A positive association between D allele and high blood pressure, atherosclerosis, coronary artery disease, stroke, diabetic nephropathy and Alzheimer’s disease has been extensively reviewed [24]. The molecular underpinning of these diseases is multi-factorial and complex, and the presence of the ACE deletion polymorphism may contribute to influence disease pathology. Indeed, to date, there is distinct lack of consensus studies linking the presence of ACE deletion polymorphism to disease causality. Nevertheless, the increase in levels of ACE in individuals with the ID and DD genotypes and potential augmentation of the RAS system and associated signalling cascades can influence pathways to influence disease pathology [25] (Fig. 1b). Indeed, increased levels of ACE and Ang-II have been implicated in the pathophysiology of lung (pulmonary hypertension, pulmonary fibrosis, acute lung injury and acute respiratory distress syndrome [26, 27]) and kidney disease (chronic kidney disease, diabetic nephropathy [28, 29]). In the African American population, the deletion polymorphism is associated with increase in systolic blood pressure, hypertension and altered vascular reactivity with potential impact on cardiovascular disease [30–32].

A subset of individuals with a positive diagnosis of COVID-19 infection have rapid progression of lung dysfunction leading to acute respiratory distress with potential need for ventilatory support [2, 3]. Presence of ACE insertion/deletion (I/D) polymorphism is associated with susceptibility and is an independent risk factor for mortality in patients with acute respiratory distress syndrome (ARDS) [33, 34]. Of the three ACE polymorphisms, there is positive association with frequency of the DD allele and incidence of ARDS, increased fatality and a prognostic factor of outcomes [35–37]. Further, the DD genotype is usually associated with higher ACE levels relative to other genotypes and with increased mortality in acute lung injury (ALI)/ARDS patients [38, 39]. Elevated levels of ACE have been observed in the bronchoalveolar fluid of individuals with ARDS [28]. Although decreases in circulating ACE have been reported in ARDS patients [40], this might be a consequence of the progressive damage to lung tissue as increased levels of ACE are evident in the bronchoalveolar fluids of individual with ARDS [40]. The positive relationship between DD genotype and ALI/ARDS and the corresponding increase in ACE levels suggest the potential involvement of increased Ang-II in the etiopathology of ARDS. During the avian (H7N9) flu infections, approximately 70% of patients developed ARDS [41]. In a subset of infected patients, increase in plasma Ang-II levels was linked to severity and fatal outcomes [41].

Within the COVID-19-infected population, there is increased incidence of kidney injury associated with higher mortality rates [42, 43]. Chronic kidney disease (CKD) is associated with severity of COVID-19 infection [44]. Interestingly, both ACE and ACE2 expressions in the kidneys are predominant in the proximal tubules with minor expression in the glomerular apparatus [45]. The balance between Ang-II and Ang (1–7) affects renal RAS to maintain balance of kidney functions; imbalance of the ratio results in kidney disease [46–48]. Chronic kidney disease is characterized by decreases in cardiac and renal ACE2 in human [49]. Diabetic nephropathy (a CKD) is characterized by decrease in ACE2, increased ACE and Ang-II-mediated tubular and glomerular damage as a result of renal RAS activation [28, 29]. Based on these studies, the ability of COVID-19 to bind and decrease ACE2 in target tissues is most likely responsible for the observed increase in blood urea nitrogen, proteinuria and hematuria associated with kidney damage [49]. Thus, COVID-19-associated decrease in ACE2 most likely results in disruption of the ACE/ACE2 balance in the kidney leading to sustained activation of ACE and Ang-II activities and kidney damage. ACE insertion/deletion polymorphism is also associated with diabetic kidney disease, the frequency of DD and ID genotype distribution being higher compared with non-diabetic kidney disease cohorts, leading to functional decline [50, 51]. The above observations suggest that presence of the DD genotype of ACE in patients with COVID-19 infection may be associated with severe respiratory distress compared with the other genotypes.

Multiple studies have reported on the prevalence of ACE I/D polymorphism, specifically the ID and DD polymorphism in increasing levels of ACE and Ang-II, which could in part influence susceptibility to underlying pathologies considered high risk for COVID-19 infections, progressive organ dysfunction and poor outcomes. Thus, presence of ID and DD polymorphism by itself is a potential underlying risk factor associated with severity and outcomes in individuals with positive diagnosis of COVID-19 infection [20, 21].

## ACE-2 Inhibition by COVID-19: Increased RAS Activity

The proteolytic cleavage of Ang-II by ACE2 to generate Ang (1–7) represents a major event leading to the physiological inactivation of Ang-II function. Thus, in patients with active COVID-19 infections, decrease in ACE2 expression/activity should most likely lead to sustained ACE-mediated generation of Ang-II and downstream signalling deleterious to organ functions including that of lung, kidney and heart [52]. Although the status of circulating and lung ACE levels in COVID-19 patients is unclear, the ability of SARS-CoV-2 binding specifically to ACE2 decreases its expression and activity suggesting upregulation of ACE/Ang-II-mediated activities. This is consistent with the observation that knockdown of ACE2 is associated with severe ARDS in multiple rodent models compared with corresponding wild-type controls [18]. Loss of ACE2 expression in mutant mice is associated with worse lung function and characterized by increases in vascular permeability, lung oedema and neutrophil accumulation [18]. Interestingly, reduced plasma levels of ACE2 are also observed within populations of African descent including African Americans, specifically in individuals with pre-hypertensive status, diabetes and renal disease [53, 54]. Administration of a catalytically active recombinant ACE2 protein improved symptoms of acute lung injury in ACE2 knockout and wild-type mice [55]. In a pilot clinical investigation, administration of recombinant human ACE2 (APN311) in patients with acute respiratory distress was associated with rapid decrease in Ang-II level and did not significantly influence oxygenation indices in the treated population compared with placebo-controlled group [56]. The recombinant human ACE2 is undergoing renewed clinical testing in the COVID-19 patient population to investigate clinical outcomes [52].

## ACE2 inhibition by COVID-19 Plus ACE D Polymorphism: Synergized RAS—Rationale for Use of ACE-I and ARBs in Clinical Management

SARS-CoV-2 binding to ACE2 results in reduction of protein expression, activity and ability to generate anti-inflammatory signalling, all of which contribute to a pro-inflammatory phenotype due to presence of ACE activity and Ang-II signalling (Fig. 1c). Presence of ACE D polymorphism increases ACE levels and Ang-II leading to pro-inflammatory phenotype and is associated with disease susceptibilities considered high risk for COVID-19 infections. Recently, it was proposed that reduced plasma levels of ACE2 in individuals of African descent most likely lowers potential for COVID-19 infection [57]; the overall outcomes in individuals with presence of ACE deletion polymorphism after infection with COVID-19 most likely leads to exacerbation of comorbidities and overall deleterious outcomes. Based on the described biological consequence of COVID-19 infections on the RAS system, treatment with ACE-I and ARBs should be associated with improved outcomes within the overall COVID-19 patient cohorts. Indeed, several meta-analyses provide preliminary support for the potential benefits of the use of ACE-I/ARBs in management of COVID-19 infections.

In a multicenter study of 1128 adult patients with hypertension with positive COVID-19 diagnosis, in-patient use of ACE-I/ARB was associated with reduced risk of mortality from all causes when compared with patients not treated with the medications [58]. Recent publications further highlight the use of ACE-I and ARBs in providing cardiovascular and renal benefits to patients with COVID-19 diagnosis [59, 60]. In a meta-analysis, patients treated with ACE-I/ARBS had 44% reduction in odds of developing severe disease and death compared with patients not treated with ACE-I/ARBs [61]. These studies provide rationale for investigation into the utility of ACE-I/ARBs in the ethnic population with known prevalence of ACE deletion polymorphisms in an effort to mitigate severity and improve outcomes in response to COVID-19 infections.

## Use of ACE-I/ARBs in Ethnic Population with Increased Prevalence of ACE D Polymorphism for Management of COVID-19

ACE is a multi-functional, relatively non-specific peptidase enzyme with a wide range of substrate specificities that impact physiological pathways in influencing blood pressure, haematopoiesis, hormone regulation, renal function and immune responses. The specificity of hypertension and cardiovascular disease as underlying causes for severity of COVID-19 infection, the inherent role of ACE-mediated generation of Ang-II and downstream signalling to potentially exacerbate inflammation and organ damage along with genotypic impact on ACE status provide compelling support of the use of ACE-I and ARBs in the clinical management of patient with positive diagnosis of COVID-19.

The biological impact of the presence of deletion polymorphism of ACE in individuals with COVID-19 infection provides a significant rationale for serious consideration of short-term use of ACE-I and/or ARBs in patients without underlying issues with blood pressure or cardiovascular disorder. The guidance statement issued by the Heart Failure Society of America (HFSA), the American College of Cardiology (ACC) and American Heart Associated (AHA) states that in the absence of favourable or detrimental effects of ACE-I and ARBs in the COVID-19 setting, the recommendation is to not arbitrarily or pre-emptively discontinue these agents in patients currently on the medication as standard of care (acc.org). Indeed, both ACE-I and ARBs have been extensively used in conditions ranging from hypertension, congestive heart failure, prevention of kidney failure and other indications. Both classes of drugs have extensive use history, understanding of safety, tolerability, efficacy, adverse events profile and drug interactions. The significant genetic, scientific and clinical data supporting a potential role for increased ACE levels and associated Ang-II effect in target organs provides compelling argument for use of ACE-I and ARBs in the clinical management of patients with COVID-19 infections to improve outcomes. High salt sensitivity–associated low plasma renin activities are responsible for the attenuated blood pressure–lowering response of ACE-I in the African American population [62]. However, this particular phenomenon might be of potential advantage in dosing and management of severity of COVID-19-associated morbidities in African American and other ethnic populations with ACE deletion polymorphism.

In summary, this study describes the biological relevance of genetic polymorphism of ACE deletion with higher prevalence in certain ethnic populations including African Americans in context of COVID-19 infection and rationale for the use of ACE-I/ARBs for therapeutic management of severity of morbidity and improving outcomes associated with COVID-19.

## Data Availability

All data obtained from public resources and referenced in the manuscript.
